# Herbal Formula HT048 Attenuates Diet-Induced Obesity by Improving Hepatic Lipid Metabolism and Insulin Resistance in Obese Rats

**DOI:** 10.3390/molecules21111424

**Published:** 2016-10-25

**Authors:** Yoon Hee Lee, Bora Jin, Sung Hyun Lee, MiKyung Song, HyeonHui Bae, Byung Jae Min, Juyeon Park, Donghun Lee, Hocheol Kim

**Affiliations:** 1 Korea Institute of Science and Technology for Eastern Medicine (KISTEM), NeuMed Inc., Seoul 130-701, Korea; eiyoon88@neumed.co.kr (Y.H.L.); jbr0627@neumed.co.kr (B.J.); lsh@neumed.co.kr (S.H.L.); ssong8230@gmail.com (M.K.S.); qogusgml87@neumed.co.kr (H.H.B.); agena@neumed.co.kr (B.J.M); saeim@neumed.co.kr (J.P.); 2 Department of Herbal Pharmacology, College of Korean Medicine, Kyung Hee University, Seoul 130-701, Korea; allstart2925@naver.com

**Keywords:** anti-obesity, insulin resistance, HT048, *Citrus unshiu*, *Crataegus pinnatifida*

## Abstract

It is well established that obesity causes a variety of chronic diseases such as cardiovascular diseases and diabetes. Despite the diligent scientific efforts to find effective ways to lower the level of obesity, the size of obese population grows continuously around the world. Here we present the results that show feeding diet containing HT048, a mixture of the extracts of *Crataegus pinnatifida* leaves and *Citrus unshiu* peel, two of the well-known traditional herbal medicines in Eastern Asia, decreases obesity in rats. We fed rats with five different diets for 10 weeks: chow diet (STD), high-fat diet (HFD), high-fat diet with 0.04% orlistat, a drug to treat obesity (HFD + Orlistat), high-fat diet with 0.2% HT048 (*w*/*w*; HFD + 0.2% HT048), and high-fat diet with 0.6% HT048 (*w*/*w*; HFD + 0.6% HT048). It was found that both body and total white adipose tissue weight of HT048 groups significantly decreased compared to those of the HFD group. Moreover, HT048 decreased serum insulin levels in HFD-fed obese rats. At the molecular level, HT048 supplementation downregulated genes involved in lipogenesis, gluconeogenesis, and adipogenesis, while the expression level of β-oxidation genes was increased. Supplementation-drug interactions are not likely as HFD and HT048-containing diet did not significantly induce genes encoding CYPs. Collectively, this study suggests that HT048 taken as dietary supplement helps to decrease obesity and insulin resistance in HFD-fed obese rats.

## 1. Introduction

Obesity induced by excessive consumption of foods rich in fat and carbohydrates is a growing global problem because obesity increases the lifetime risk of cardiovascular diseases, hypertension, diabetes, and certain cancers, all of which are associated with hyperglycemia, insulin resistance, high cholesterol, and fatty liver [1,2]. World Health Organization (WHO) classified obesity as a disease [3]. Obesity is characterized by excessive adiposity that creates an imbalance between energy intake and energy expenditure. Adipose tissue is closely associated with insulin sensitivity and energy homeostasis [4,5]. White adipose tissue (WAT) stores excessive energy as triglycerides and mobilizes energy in the form of free fatty acids. Adipose tissue is composed of adipocytes and it secretes a number of adipokines that mediate lipid metabolism, systemic inflammation, and insulin sensitivity. These abnormal metabolic conditions can progress to states of obesity-associated diseases [6].

Current methods to treat obesity include diet, exercise, and drug therapy [7]. Orlistat, Lorcaserin, and Phentermine/topiramate are oral anti-obesity drugs approved by the US Food and Drug Administration (FDA). Orlistat inhibits lipase and absorption of fat in the digestive tract, while Lorcaserin and Phentermine/topiramate control appetite and food intake behavior, all of which significantly reduce body weight, total cholesterol, and triglycerides. However, recent studies show adverse effects of these drugs such as steatorrhea, nausea, dizziness, and insomnia [8]. Several anti-obesity drugs have been found to cause adverse side-effects as well and withdrawn from the market. Novel therapeutic strategies with minimal side effects are urgently needed.

An increasing number of natural products including *Garcinia Gambogia*, white bean extract, bitter orange, and green coffee are reported to decrease the level of diet-induced obesity or hyperglycemia in animal models, making natural products a promising pool of candidates for drug or dietary supplements to treat obesity [9]. *Citrus unshiu* (*Ci. unshiu*), is one of the most commonly consumed citrus species in Korea, is a sweet-tasting, seedless, and easy-peeling fruit. Its peel has been used as traditional herbal medicine to improve bronchial and asthmatic conditions or blood circulation in Korea, China, and Japan [10]. Previous studies demonstrated that *Ci. unshiu* peel provides health benefits such as antioxidant, anti-inflammatory, anti-allergic, anticancer, and cardiovascular protective [11,12]. *Ci. unshiu* peel contains abundant functional compounds, particularly naringin, hesperidin, and nobiletin [13]. *Crataegus pinnatifida* (*Cr. pinnatifida*) has been traditionally used for traditional medicine in Asia (Korea, China, and Japan) [14]. The main bioactive components in *Cr. pinnatifida* are chlorogenic acid, epicathchin, vitexin, rutin, and hyporoside and these components are good treatment for hypertension, cardiovascular, antioxidative, atherosclerosis, and hyperlipoidemia diseases [15,16,17].

In the previous studies, we demonstrated anti-obesity activity of the extracts of the peel of *Ci. Unshiu* and the fruit and the leaves of *Cr. Pinnatifida* fruit in high-fat diet (HFD)-induced obese rats, reducing body weight and serum lipid concentration [18]. When mixed and fed, HT048, the mixture of the extracts of *Ci. unshiu* peel and *Cr. pinnatifida* leaves, even resulted in lowering the level of obesity in rats [19]. In this study, we carried out a series of experiments to further our understanding in the mechanism underlying anti-obesity activity of HT048. In addition, we tested whether HT048 exerts promoting effect on thermogenesis and glucose metabolism. Cytochrome P450 superfamily (CYPs) are one of the most important drug-metabolizing enzymes and it was modulated by dietary supplementation and foods [20]. Therefore, it was investigated whether HT048 increases or reduces the transcriptional level of genes encoding the CYPs in rats.

## 2. Results

### 2.1. Food Intake, Body Weight, and Weight Gain

To evaluate whether HT048 has an anti-obesity effect, we measured the body weight of rats every week during the 10-week feeding period. There was no significant difference in food intake among HFD-fed groups (Table 1). By the end of the feeding period, final body weight and body weight gain of the HFD group was significantly higher than those of the STD group (*p* < 0.0001 and *p* < 0.0001). HT048 supplementation significantly reduced final body weight and weight gain in HT048 groups compared to those of HFD group.

### 2.2. Changes in Organ Weights

**Figure 1:**
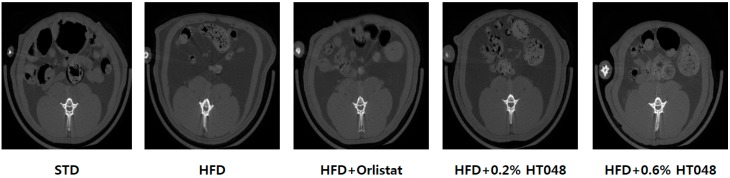
Effect of HT048 on abdominal fat accumulation in HFD-fed obese rats. Representative microCT scanning images of abdominal WAT for each group are shown. White, gray, and black parts roughly indicate bone, muscle or viscera, and fat, respectively.

Table 1 depicts the weight of liver, spleen and WAT (epididymal, mesenteric, and perirenal). The weight of liver and WAT of HFD group was significantly higher than that of STD group. Significant difference was also found in the liver weight among HFD group, HFD + 0.2% HT048 and HFD + 0.6% HT048 groups. As expected, diet with HT048 supplementation significantly decreased visceral fat mass including the weight of epididymal, mesenteric, and perirenal WAT after 10 weeks of administration, compared to HFD group (*p* < 0.0001, *p* < 0.0001 and *p* < 0.0001). However, there was no significant difference in these parameters between HFD + 0.2% HT048 and HFD + 0.6% HT048 groups. Furthermore, in vivo whole-body scans for abdominal fat deposition using microCT revealed that the abdominal fat in HFD group was more accumulated than in STD group indicating positive effect of HT048 in reducing abdominal fat accumulation (Figure 1).

### 2.3. Biochemical Analysis of Serum Samples

There was a considerable increase in the levels of serum insulin and glucose in HFD-fed rats compared with the standard chow diet fed rats (*p* = 0.0095 and *p* = 0.0003). Serum insulin levels significantly decreased in HT048 groups compared with the HFD group. However, there was no significant difference in the level of fasting glucose among the HFD and HT048 groups (Table 2).

### 2.4. Expression of Genes Involved in Lipogenesis, β-Oxidation-Related Genes, and Gluconeogenesis in the Liver

**Figure 2:**
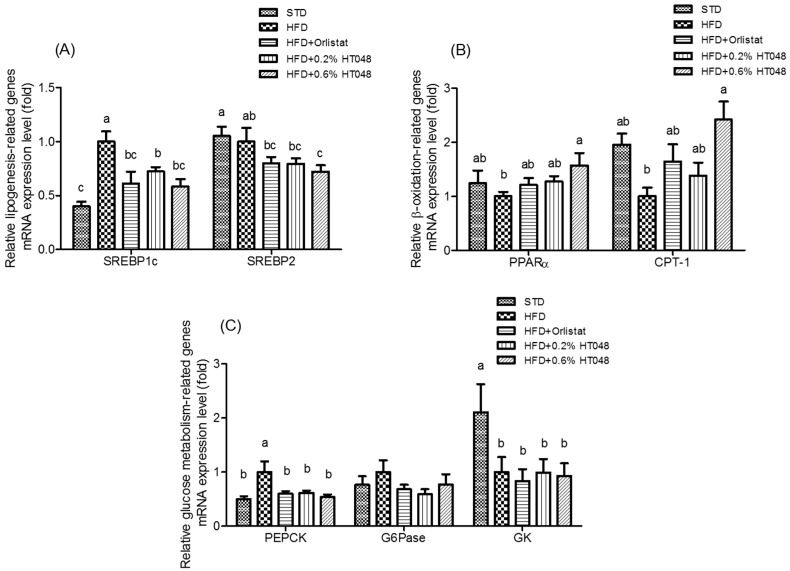
Effect of HT048 on mRNA expression of genes related to hepatic lipogenesis (**A**); β-oxidation (**B**); and glucose metabolism (**C**) in HFD-fed obese rats. Values are mean ± SD (*n* = 12 for each group). Data were analyzed by one-way analysis of variance (ANOVA) followed by Duncan’s multiple range test. abc Means not sharing common letters are significantly different among the groups at *p* < 0.05.

The transcriptional level of genes involved in lipogenesis and β-oxidation was determined (Figure 2). The expression level of two lipogenic genes, sterol regulatory element-binding protein 1c (SREBP 1c) and SREBP2, was significantly lower in HFD+0.6% HT048 group than that of HFD group (*p* < 0.0001 and *p* = 0.0279, respectively, Figure 2A). Expression of β-oxidation-related genes, peroxisome proliferator-activated receptor α (PPARα) and carnitine palmitoyltransferase 1 (CPT-1), in HFD group was decreased compared to that of the STD group (Figure 2B). The level of CPT-1 expression was significantly higher in HFD + 0.6% HT048 group compared to that of HFD group (*p* = 0.0043). Moreover, the expression of PPARα mRNA was increased by HT048 in HFD-fed rats, although there was no significant difference. We also determined the expression level of gluconeogenesis-related genes, glucokinase (GK), phosphoenolpyruvate carboxykinase (PEPCK), and glucose-6-phosphatase (G6Pase) in the liver (Figure 2C). HFD-fed rats exhibited a decreased level of the expression of GK compared to STD rats (*p* = 0.0385). However, there was no significant difference in the expression of GK among the HFD group and HT048 groups. The expression level of G6Pase in HT048 groups is somewhat lower compared to that of HFD group, but the difference was not statistically significant (*p* = 0.4494). HFD treatment upregulated PEPCK compared with the STD group and the level of PEPCK expression was significantly lower after 10-week diet supplementation with HT048 (*p* = 0.0057).

### 2.5. Expression of Fat Accumulation-Related and Inflammatory Genes in WAT

**Figure 3:**
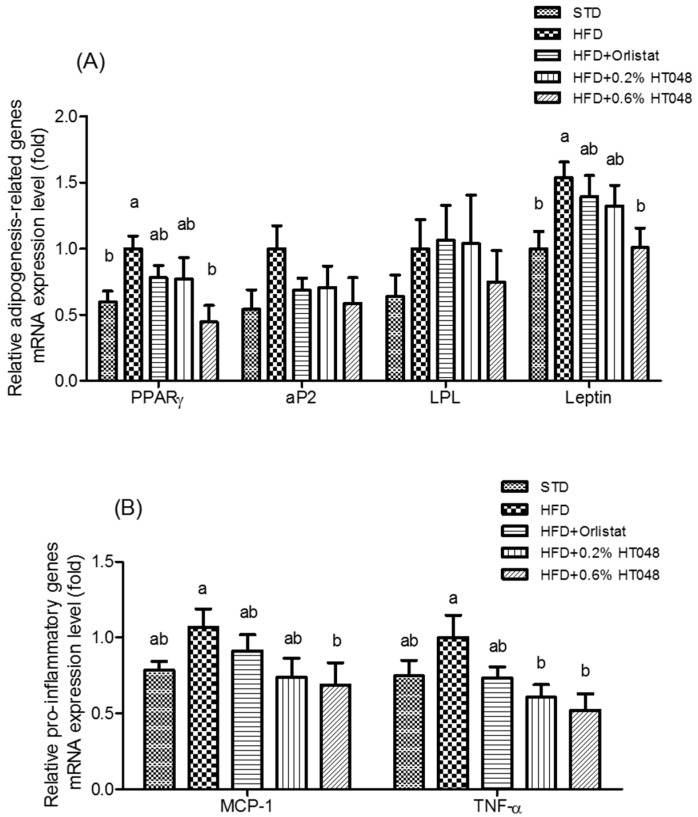
Effect of HT048 on mRNA expression of genes related to WAT adipogenesis (**A**) and pro-inflammatory (**B**) in HFD-fed obese rats. Values are mean ± SD (*n* = 12 for each group). Data were analyzed by one-way analysis of variance (ANOVA) followed by Duncan’s multiple range test. ab Means not sharing common letters are significantly different among the groups at *p* < 0.05.

In order to investigate the effect of HT048 on adipocyte differentiation and fat accumulation, we assessed changes in the expression level of key genes to adiposity including peroxisome proliferator-activated receptor gamma (PPARγ), adipocyte protein 2 (aP2), lipoprotein lipase (LPL), and leptin in WAT (Figure 3A). As expected, HFD group showed the highest levels of expression of PPARγ and leptin and 0.6% HT048 supplementation significantly reduced PPARγ and leptin mRNA expression (*p* = 0.0249 and *p* = 0.0353). However, there was no significant difference in the transcriptional level of aP2 and LPL among STD, HFD, and HT048 groups (*p* = 0.2643 and *p* = 0.6652, respectively). Moreover, we studied whether HT048 influenced the expression of adipose tissue inflammatory genes such as monocyte chemoattractant protein-1 (MCP-1) and tumor necrosis factor alpha (TNF-α). The level of expression of TNF-α was significantly decreased by HT048 supplementation (*p* = 0.0322, Figure 3B). The expression of MCP-1 mRNA was decreased by HT048 in HFD-fed rats, but there was no significant difference (*p* = 0.1530).

### 2.6. Expression of Genes Encoding CYP Enzymes

**Figure 4:**
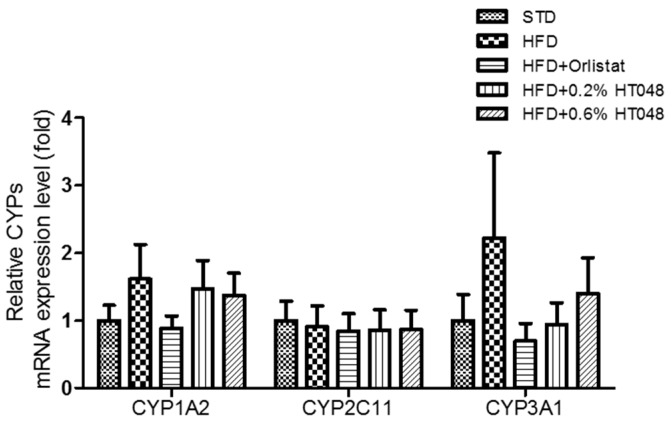
Effect of HT048 on mRNA expression of hepatic CYP genes in HFD-fed obese rats. Values are mean ± SD (*n* = 12 for each group). Data were analyzed by one-way analysis of variance (ANOVA) followed by Duncan’s multiple range test.

Finally, we measured the expression level of cytochrome P450 1A2 (CYP1A2) (*p* = 0.5491), cytochrome P450 2C11 (CYP2C11) (*p* = 0.9959), and cytochrome P450 3A2 (CYP3A2) (*p* = 0.4892) in the liver to investigate the effect of HT048 on three major CYP isozymes. There was no difference among groups for any CYP genes tested (Figure 4).

## 3. Discussion

Excess caloric intake and lack of energy expenditure are generally accepted to disrupt hepatic lipid metabolism and induce obesity and insulin resistance [21]. HT048 is an herbal formula containing extracts of *Cr. pinnatifida* leaves and *Ci. unshiu* peel as main ingredients. In this study, we presented results showing that orally administrated HT048 decreases obesity in rats induced by HFD.

Several studies have demonstrated that the individual ingredients of HT048, *Cr. pinnatifida* and *Ci. unshiu*, improve lipid and glucose metabolism in HFD-fed obese animals [10,22,23], which led us to investigate the effect of their mixture, HT048, on obesity, hyperglycemia, hyperlipidemia, insulin resistance, and inflammation in HFD-fed obese rats. First, we showed that the body weight and WAT of HFD-induced obese rats were significantly reduced without any change in food intake only by feeding rats with HT048-supplemented diet. Besides, we determined the abdominal fat deposition using microCT, but did not quantify. The reason why we could not measure all the animals with microCT is that microCT might affect the parameters because rats should be given an anesthesia for three hours and it is not possible to measure the volumes of epididymal fat, mesenteric fat, and perirenal fat, respectively. Therefore, we selected the rats that were closest to the average body weight of each group for the representative pictures and measured the weights of epididymal fat, mesenteric fat, and perirenal fat respectively. Excessive visceral fat is strongly associated with metabolic disturbance [24]. HT048 reduced liver fat as well. Increased liver weight of HFD-fed obese rats, the sign of hepatic lipid accumulation and development of fatty liver in HFD-fed animals [25,26] was significantly reversed by supplementation of HT048. The spleen is an important organ in the immune system. The spleen can enlarge by performing its functions in response to another medical condition. Splenomegaly is also found among people with obesity because obesity is a risk factor for deteriorating cellular immune functions by decreases in both T lymphocyte response and B lymphocyte response [27]. In rats, it is also reported that abnormal enlargement of spleen is induced by a high-fat diet [28]. In the present study, spleen weight was significantly decreased in the HFD+HT048 group compared with the HFD group but there was no statistical difference between HFD+HT048 group and STD group. It means HT048 may restore spleen weight of rats fed high-fat diet. These results indicate that ingestion of HT048 inhibits weight gain and accumulation of visceral fat in HFD-fed obese rats without affecting appetite and HT048 can be considered as attenuating the immune dysfunction induced by obesity.

Our previous studies showed that HT048 improved the level of serum triglyceride (TG), total cholesterol (TC), low-density lipoprotein (LDL-C), and high-density lipoprotein (HDL-C) in HFD-induced obese rats [18,19]. In this study, we expanded our research scope and looked into the effect of HT048 on sugar metabolism. HT048 decreased not only the level of serum lipids as we previously reported, but also the concentration of serum insulin. Insulin is a key regulator of lipid and glucose metabolism; elevated plasma insulin levels may incur obesity and insulin resistance [29,30], and impaired insulin sensitivity often accompanies increased glucose production and lipolysis and reduced glucose uptake in the liver [21]. HT048 orally administered with HFD for 10 weeks did not lead to significant changes in fasting blood glucose level in the test animals. However, in HT048 groups compared to HFD groups, the expression level of hepatic PEPCK gene was markedly downregulated, indicative of positive effect of HT048 on the gluconeogenesis in obese rats. Expression of another gluconeogenesis gene, G6Pase, was also lower in HT048 groups than in HFD, although the difference was not statistically significant. Overexpression of hepatic gluconeogenesis genes including PEPCK and G6Pase induced insulin resistance and diabetic phenotype in rodents [31]. GK plays a major role in glucose homeostasis, and insulin resistance reduces GK activity due to GK gene expression induced by insulin [32]. Therefore, despite no significant change in the blood glucose level, these results suggest that HT048 may help maintain blood glucose level by suppressing the expression of gene-related gluconeogenesis and inducing the expression of genes-related glucolysis. Moreover, the serum insulin level increased by HFD was reduced by HT048 supplementation in obese rats. It appears that HT048 aids in maintaining insulin sensitivity in obese rodents.

Insulin resistance, a hallmark of obesity, induces lipogenic transcription factors such as SREBP and carbohydrate response element binding protein (ChREBP). Lipogenesis is an insulin- and glucose-dependent process, where excessive carbohydrates converse into fatty acids [33]. In mammals, there are two SREBP genes (SREBP-1 and -2). SREBP1 regulates fatty acid metabolism, whereas SREBP2 regulates cholesterol metabolism [34]. SREBP1c, is a key lipogenic transcription factor, mRNA regulates transcriptional regulation of lipogenic enzymes such as FAS and SCD [35,36]. PPARα is abundantly expressed in tissue with high rates of fatty acid β-oxidation and regulates the steps in the downstream such as CPT-1, a key enzyme to fatty acid β-oxidation in the liver [37]. HFD increases hepatic lipogenesis and decreases fatty acid oxidation [38] which was completely reversed when HFD was supplemented with HT048 and fed to rats; HT048 supplementation reduced the expression of hepatic lipogenic genes and increased the expression of hepatic β-oxidation-related genes. These results suggest that the effect of HT048 on hepatic lipid accumulation might be regulated by suppression to precursor cells at the onset of obesity.

The accumulation of WAT could lead to increased flux of free fatty acids to the liver, and elevated free fatty acids could increase TG content in the liver and decrease insulin clearance in the serum [39]. Several studies describe that the expression of adipogenic genes including PPARγ, LPL, aP2, and leptin may lead to higher visceral fat in obese animals [40,41]. PPARγ is an important transcription factor that increases adipogenesis, which includes pre-adipocyte proliferation and adipocyte differentiation by activating aP2 and LPL [22]. LPL regulates the entry of TGs into adipose tissue and muscles and aP2 is a member of the cytoplasmic fatty acid-binding protein family [42,43]. In the previous studies, PPARγ mRNA expression regulates its target genes such as LPL and aP2 in rodents [19,36]. In addition, leptin is one of the adipokines that stimulated adipogenesis in HFD-fed mice [44]. Consistent with the above observations, HT048 suppressed the expression level of PPARγ and leptin. WAT is composed primarily of mature adipocytes, which are generated through the proliferation of preadipocytes [45]. Chronic inflammation is a characteristic of obesity that is caused because mature adipocytes inducing expression of pro-inflammatory cytokines genes including MCP-1 and TNF-α [46]. We observed that HT048 supplementation suppresses pro-inflammatory cytokines, MCP-1, and TNF-α. MCP-1 and TNF-α contribute to monocyte recruitment intro WAT and pathogenesis of obesity-linked complications in WAT, respectively [46]. Our results suggest that decreased WAT mass in HT048 groups is strongly associated with downregulation of adipogenesis genes and pro-inflammatory cytokines.

Cytochrome P450 monooxygenases are a superfamily of heme-thiolate enzymatic proteins involved in the activation and metabolism of drugs [47]. Induced level of CYPs genes indicates possible side effects induced by herb-drug interactions. Among numerous CYP isozymes identified to date, CYP1A2, CYP2C11 (corresponding to human CYP2C9), and CYP3A1 (corresponding to human CYP3A4) play the dominant roles [48]. Therefore, we determine the effects of HT048 on the CYP1A2, CYP2C11, and CYP3A1 mRNA expression in liver of HFD-induced obese rat. There was no significant difference in CYP1A2, CYP2C11, and CYP3A1 among all groups. CYPs may be particularly vulnerable to modulation by dietary supplements and foods, including fruits, vegetables, herbs, spices, and teas [20]. Several studies demonstrated that high-fat diet, sex, hormonal imbalance, and so on influence the activity and expression of hepatic CYPs in rodents [47,49,50]. Thus, more research is needed to accurately determine pharmacokinetic effect of HT048 in standard rodents fed a normal diet.

In summary, HT048 not only reduces body weight and lipid accumulation but also improves insulin sensitivity in HFD-fed obese rats. HT048 upregulates β-oxidation related genes and downregulates enzyme activities involved in gluconeogenesis, lipogenesis, adipogenesis, and pro-inflammation in liver and adipose tissue. Based on these findings, HT048 may be useful for preventing or retarding the development of obesity and its metabolic complications such as dyslipidemia, hepatic steatosis, and insulin resistance.

## 4. Materials and Methods

### 4.1. Preparation of HT048

Dried *Cr. Pinnatifida* leaves and dried *Ci. unshiu* peel were purchased from Anhui Jinzhai Qiaokang (Jinzhai, China) and Dong Kwang Pharm (Seoul, Korea), respectively. The samples were identified by Dr. Hocheol Kim, and the voucher specimens (#HP570 and #HP013) deposited at the Department of Herbal Pharmacology, College of Korean Medicine, Kyung Hee University (Seoul, Korea). The dried *Cr. pinnatifida* leaves and dried *Ci. unshiu* peel were individually extracted in 30% ethanol (*v*/*v*) for 6 h at 85 °C in a reflux apparatus. The extracts were filtered concentrated under reduced pressure and spray-dried (SD) with 33.96% dextrin for *Ci. unshiu* and 18.60% for *Cr. pinnatifida*. The extraction yields of *Ci. unshiu* peel and *Cr. pinnatifida* leaves (%) were 21.3% and 19.02%, respectively. The resultant SD powdery extracts of *Ci. unshiu* and *Cr. Pinnatifida* were mixed at the ratio of 53:43, which is HT048.

### 4.2. HPLC Analysis of HT048

**Figure 5:**
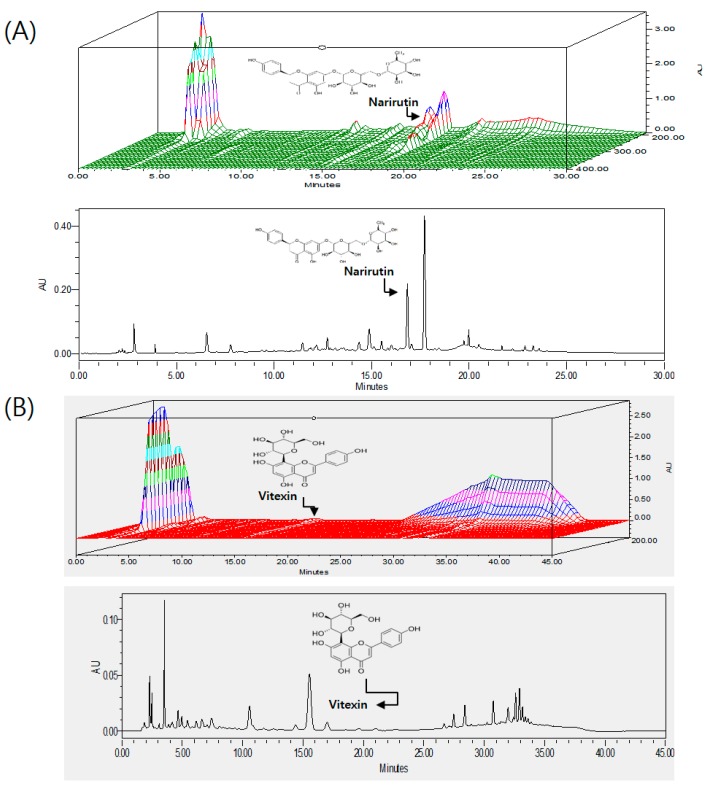
3-D HPLC chromatogram of HT048, a blend of two herbal extracts (**A**) *C. unshiu* peel of narirutin; (**B**) *C. pinnatifida* leaves of vitexin.

Quality of HT048 was controlled by measuring the amount of vitexin and narirutin, marker compounds of *Cr. pinnatifida* and *Ci. unshiu*, respectively. As *Cr. pinnatifida* contains vitexin derivatives such as vitexinrhamnoside and vitexin glucoside that tend to degrade over time, the labile vitexins were acid-hydrolyzed into vitexin, which was determined as total vitexin using HPLC. HT048 samples were used for further experiments only when the concentrations of narirutin and total vitexin were 5.56 ± 0.14 mg/g and 3.31 ± 0.05 mg/g, respectively. For HPLC analysis, 2 g of HT048 SD powder was hydrolyzed in 1 M HCl in water (100 mL). The hydrolysis was performed in triplicate. The acid-hydrolyzed sample was refluxed at 100 °C for 1 h, allowed to cool to room temperature, diluted up to 200 mL and sonicated for 5 min. The extract was filtered through a 0.45 μm filter prior to injection to a Waters HPLC system (Waters, Milford, MA, USA) comprising a Waters 1525 pump, a 2707 autosampler, and a 2998 PDA detector. The chromatic separation was achieved at 35 °C on a Waters Sunfire™ C18 column (250 mm × 4.6 mm I.D., 5 μm particle size). A binary solvent system was employed consisting of 0.05% phosphoric acid in water as solvent A and tetrahydrofuran-acetonitrile (20:3, *v*/*v*) as solvent B. The gradient program to analyze vitexin was 0–20 min with 20% solvent B, 20–30 min with 20%–50% solvent B, 30–35 min with 50% solvent B, 35–37 min with 50%–20% solvent B, 37–45 min with 20% solvent B. For narirutin, mobile phases A and B were 0.05% phosphoric acid in water (*v*/*v*) and acetonitrile, respectively, and the gradient program was 0–15 min with 5%–30% solvent B, 15–20 min with 30%–80% solvent B, 20–25 min with 80%–5% solvent B, 25–30 min with 5% solvent B. The flow rate was 1.0 mL/min, and the injection volume was 10 μL. The sample was injected twice to analyze each marker compound, and the eluents from the column were monitored at 284 nm for narirutin and 330 nm for vitexin. A 3-D chromatogram of HT048 is given in Figure 5.

### 4.3. Animal Experiments

Three week old Sprague-Dawley male rats were supplied by Samtako (Gyeonggi-do, Korea). Rats were acclimated in polycarbonate cages and reared at 23 ± 1 °C, 55%–60% of relative humidity with a 12/12 h light/darkness cycle on commercial standard chow diet (Samtako, Korea) and water ad libitum. After seven days for adaptation, all SD rats of four weeks of age were then randomly divided into five groups as follows: Group 1, STD group that received only chow diet (*n* = 12); Group 2, HFD group that received 60% HFD control (D12492, Research Diets Inc., New Brunswick, NJ, USA); Group 3, HFD + Orlistat group that was fed with HFD containing 0.04% orlistat (*w*/*w*); Group 4, HFD + 0.2% HT048 group that was fed with HFD containing 0.2% HT048 (*w*/*w*); Group 5, HFD +0.6% HT048 group that was fed with HFD containing 0.6% HT048 (*w*/*w*). They were provided with fresh food and distilled water every 2–3 days for 10 weeks of administration. At the end of the experiment, all rats were euthanized after 12 h of fasting. Blood samples were collected from abdominal aorta, left at room temperature for 20 min, centrifuged at 1000× *g* at 4 °C for 10 min, the separated serum was collected and stored at −80 °C until biochemical analysis. Liver and WAT samples were promptly dissected out, rinsed off with saline, weighed, and stored at −80 °C until further analysis.

### 4.4. Serum Measurements

Serum insulin concentration was determined using commercially-available enzyme-linked immunosorbent assay (ELISA) kit (EZRMI-13K, Millipore, MA, USA). Fasting glucose levels was measured using enzymatic assay kits (V-Glucose, Asan Pharm., Seoul, Korea), respectively, according to the manufacturer’s instructions.

### 4.5. Real-Time Quantitative PCR Analysis

Total RNA samples were isolated from the rat liver and adipose tissue using Trizol reagent (Invitrogen Technologies, Waltham, MA, USA). RNA samples were converted to cDNA using High Capacity cDNA Reverse Transcription kit (Applied Biosystems, Foster City, CA, USA) according to the manufacturer’s instruction. The cDNA was used for real-time quantitative polymerase chain reaction (PCR). Real-time quantitative PCR was performed on a Step One Plus Real Time PCR system (Applied Biosystems) as follows: after 10 min at 95 °C, 40 cycles of 15 s at 95 °C, and 60 s at 60 °C, followed by the melting curve for 15 s at 95 °C, gradual decrease to 60 °C in the last 60 s, then gradual increase to 95 °C for the last 15 s. The relative gene expression was normalized using the housekeeping gene. Primers were designed using nucleotide sequence and synthesized by Bioneer (Bioneer, Daejeon, Korea). The sequences of the primers used in this study are listed in Table 3. The relative fold change of gene expression was calculated using the Delta-Delta method.

### 4.6. Micro-Computed Tomography (microCT)

After 10 weeks of feeding, rats were fasted for 12 h and anesthetized with isoflurane. Each rat was laid on a scanning bed and the whole-body was scanned and its tomography was analyzed using Skyscan micro-CT system (Skyscan 1176; Skyscan, Kontich, Belgium). MicroCT was performed using the following parameters: resolution, 30 μm; voltage, 100 kV; source current, 100 μA; and rotation step, 0.5°. Transverse microCT images of the abdomen from L1 to L5 were scanned using a micro CT-scanner. Analysis of microCT images was performed using NRecon software (Skyscan v. 1.6.10.1).

### 4.7. Statistical Analysis

Statistical analysis was performed by using SAS 9.3 (SAS Institute Inc., Cary, NC, USA). All data were presented as the mean ± standard deviation (S.D.). The effects of different treatments were compared using one-way ANOVA and Duncan’ multiple test for multiple comparisons. A value of *p* < 0.05 was considered statistically significant.

## Figures and Tables

**Table 1:** Food intake, body weight, and organ weights.

Parameters	STD	HFD	HFD + Orlistat	HFD + 0.2% HT048	HFD + 0.6% HT048
Food intake (g/day)	17.90 ± 2.69 ^a^	14.39 ± 2.25 ^bc^	15.55 ± 3.74 ^b^	14.15 ± 1.96 ^c^	14.31 ± 2.32 ^bc^
Body weight (g)					
Initial weight	114.90 ± 8.05	114.93 ± 6.40	114.93 ± 9.15	114.85 ± 4.44	114.88 ± 5.94
Final weight	444.50 ± 20.64 ^c^	508.83± 17.04 ^a^	460.00 ± 32.50 ^bc^	479.92 ± 29.56 ^b^	474.75 ± 31.74 ^b^
Weight gain	329.60 ± 22.88 ^c^	393.91 ± 18.69 ^a^	345.07 ± 29.17 ^bc^	365.07 ± 30.92 ^b^	359.88 ± 31.12 ^b^
Organ weight (g)					
Liver	10.19 ± 0.83 ^b^	11.07 ± 0.51 ^a^	10.46 ± 1.13 ^ab^	10.02 ± 0.96 ^b^	9.91 ± 0.70 ^b^
Spleen	0.83 ± 0.09 ^ab^	0.86 ± 0.16 ^a^	0.81 ± 0.11 ^ab^	0.76 ± 0.09 ^b^	0.75 ± 0.10 ^b^
Epididymal	7.01 ± 1.70 ^c^	13.15 ± 2.65 ^a^	9.16 ± 2.58 ^b^	9.94 ± 1.69 ^b^	10.11 ± 2.57 ^b^
Mesenteric	2.49 ± 0.66 ^d^	6.55 ± 1.50 ^a^	3.86 ± 1.27 ^c^	5.11 ± 1.09 ^b^	5.10 ± 1.69 ^b^
Perirenal	5.63 ± 2.04 ^c^	13.01 ± 3.14 ^a^	8.63 ± 1.94 ^b^	10.55 ± 2.36 ^b^	9.61 ± 2.94 ^b^
Total WAT	15.13 ± 3.83 ^c^	32.72 ± 5.22 ^a^	21.65 ± 5.40 ^b^	25.60 ± 4.00 ^b^	24.81 ± 5.61 ^b^

Values are mean ± SD (*n* = 12 for each group). Data were analyzed by one way-analysis of variance (ANOVA) followed by Duncan’s multiple range test. ^abc^ Means not sharing common letters are significantly different among the groups at *p* < 0.05.

**Table 2:** Effect of HT048 on serum glucose, and insulin in HFD-fed obese rats.

Parameters	STD	HFD	HFD + Orlistat	HFD + 0.2% HT048	HFD + 0.6% HT048
Glucose (mg/dL)	89.29 ± 14.13 ^a^	120.29 ± 21.69 ^b^	106.64 ± 11.42 ^b^	111.29 ± 16.99 ^b^	117.46 ± 13.43 ^b^
Insulin (ng/mL)	0.62 ± 0.57 ^a^	1.42 ± 1.05 ^b^	0.48 ± 0.10 ^a^	0.74 ± 0.47 ^a^	0.96 ± 0.71 ^ab^

Values are mean ± SD (*n* = 12 for each group). Data were analyzed by one way-analysis of variance (ANOVA) followed by Duncan’s multiple range test. ^ab^ Means not sharing common letters are significantly different among the groups at *p* < 0.05.

**Table 3:** Sequences of primers used for real-time quantitative PCR analysis.

Gene	Forward Primer (5’-3’)	Reverse Primer (5’-3’)
SREBP1c	AAAACCAGCCTCCCCAGAGC	CCAGTCCCCATCCACGAAGA′
SREBP2	CACTCACGCTCCTCGGTCAC	CGGATAAGCAGGTCTGTAGGTTGG
PPARα	TGGAGTCCACGCATGTGAAG	CGCCAGCTTTAGCCGAATAG
CPT-1	TAGGACAGGCAGAAAATTGC	CAGTAGGAGCCGATTCAAAA
PEPCK	GAGGAGCTGTTCGGAATCTCTA	CCTCTCTATTTCGTAAGGGAGGTC
G6Pase	GTCAGTCTTATCCCCTACTGCCTA	CGCTGACTTGTGTCAGAGACTT
GK	GAGTCTCTCTAGCTGAGACCAACA	CAGACCTCCTTGACTAACTCAGGT
PPARγ	CCCTGGCAAAGCATTTGTAT	GGTGATTTGTCTGTTGTCTTTCC
aP2	GGCTTCGCCACCAGGAA	CCCTTCTACGCTGATGATCAAGT
LPL	CAGCAAGGCATACAGGTG	CGAGTCTTCAGGTACATCTTAC
MCP-1	AATGAGTCGGCTGGAGAACTAC	GATCTCTCTCTTGAGCTTGGTGAC
TNF-α	GGCAGGTCTACTTTGGAGTCAT	GAGTAGACGATAAAGGGGTCAGAG
CYP1A2	GTCACCTCAGGGAATGCTGTG	GTTGACAATCTTCTCCTGAGG
CYP2C11	AAAAGCACAATCCGCAGTCT	GCATCTGGCTCCTGTCTTTC
CYP3A1	CTTCACAAACCGGAGGCCTTTGGT	ATCAGGGTGAGTGGCCAGTTCTAC
